# Observation of anomalous linear photogalvanic effect and its dependence on wavelength in undoped InGaAs/AlGaAs multiple quantum well

**DOI:** 10.1186/1556-276X-9-493

**Published:** 2014-09-14

**Authors:** Laipan Zhu, Yu Liu, Hansong Gao, Xudong Qin, Yuan Li, Qing Wu, Yonghai Chen

**Affiliations:** 1 Key Laboratory of Semiconductor Materials Science, Institute of Semiconductors, Chinese Academy of Sciences, 100083 Beijing, People’s Republic of China

**Keywords:** Spectra, Anomalous linear photogalvanic effect, Optical momentum alignment effect, Momentum relaxation time

## Abstract

We observed an anomalous linear photogalvanic effect (ALPGE) in undoped InGaAs/AlGaAs multiple quantum well and studied its wavelength dependence in details. This effect is believed to originate from the optical momentum alignment effect and the inhomogeneity of light intensity. We find that the spot location with the maximum ALPGE current is wavelength independent. And the normalized ALPGE current decreasing at smaller wavelengths is attributed to the sharp decrease of the momentum and energy relaxation time. The electrical measurement of the spectra dependence of ALPGE is highly sensitive proving to be an effective method for detecting the momentum anisotropy of photoinduced carriers and band coupling.

## Background

**Figure 1:**
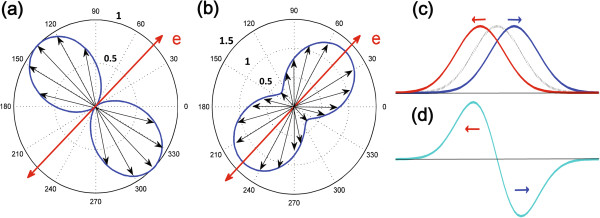
**Shape of the momentum distribution of the photoinduced electrons.** In the case of excitation by linearly polarized light, the schematic diagram for carriers under the Gaussian light spot, and the photocurrent distribution in the light spot. **(a)** and **(b)** correspond to 1hh-1e and 1lh-1e transition, respectively. In both cases the symmetry axis of the alignment is along the polarization vector, *e*, of the exciting light. The lengths of the vectors indicate the populations of the states with the corresponding momentum directions. **(c)** A schematic diagram for carriers with momentum alignment under the Gaussian light spot. During the momentum relaxation time, carriers with opposite momentum directions have a small spatial displacement. **(d)** The photocurrent distribution in the light spot is proportional to the gradient of light intensity along the momentum alignment direction.

Photocurrents induced by linearly polarized light in semiconductors have been intensively studied for decades [1-7]. Linearly polarized light can result in the linear photogalvanic effect (LPGE) [1], the photon drag effect [1], the pure spin current in a system with spin orbit interaction [2,3], and the optical momentum alignment effect [4-6] (Figure 1). Recently, an anomalous linear photogalvanic effect (ALPGE) was firstly observed in a (001)-oriented GaN-based two-dimensional electron gas (2DEG) [7]. The ALPGE occurs in (001)-oriented 2DEG with *C*_2*v*_ symmetry for normal light incidence [7], while the ordinary LPGE is forbidden by symmetry in this situation [1] (but it is not always true for other situations, such as (110)-oriented quantum wells [8]).

**Figure 2:**
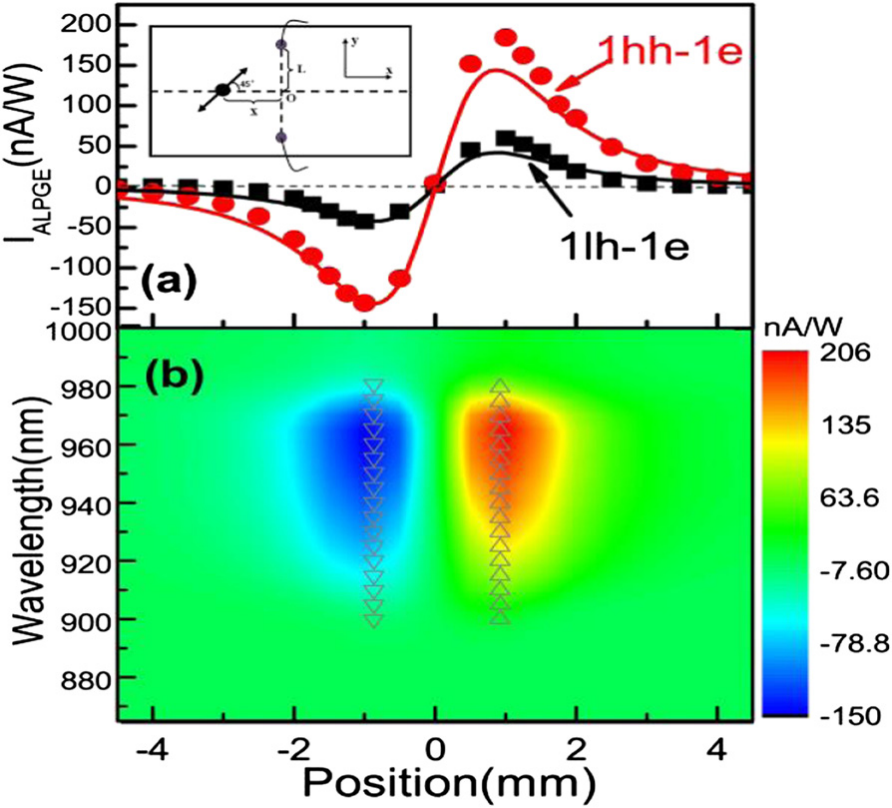
**The ALPGE current as a function of the spot location near the transitions of 1hh-1e and 1lh-1e. (a)** The ALPGE current as a function of the spot location corresponding to 1hh-1e and 1lh-1e. The two solid curves are the curves fitted by Equation (5). The inset denotes a schematic diagram of the experimental setup. **(b)** The two-dimensional colored figure denotes the ALPGE currents at different spot locations and different wavelengths (near the transitions of 1hh-1e and 1lh-1e). The uptriangles (downtriangles) mark the maximum (minimum) positions at certain wavelengths.

In the following introduction, we take the *x*, *y*, and *z* directions to be parallel to the crystal’s [110], $\left[\;1\bar{1}0\right]$, and [001] directions, respectively. For the (001)-oriented 2DEG with *C*_2*v*_ symmetry, the ordinary LPGE current can be expressed as follows:

(1) $$j_{y}\;=\;\chi_{\text{yyz}}{ê}_{x}E_{0}^{2}\text{sin}2\theta_{0},$$

where the third-rank pseudotensor *χ*_*yyz*_ is a symmetry factor; *x*,*y*, and *z* are three orthogonal directions; ***E***_0_, ê, and *θ*_0_ are the electric field amplitudes of the light, the unit vector pointing in the direction of light propagation, and the angle between the plane of polarization defined by the electric field vector and the *x* direction, respectively [1]. For normal incidence, ê is parallel to [001] crystallographic orientation and hence the current vanishes as ${ê}_{x}=0$[1]. However, under normal incidence, a sizeable charge current induced by linearly polarized light is observed with the light spot moving away from the ‘O’ point and along the *x* direction (see the inset of Figure 2a). This current corresponds to the typical characteristics of LPGE which can be described by following formula:

(2) $$j_{\text{ALPGE}}\;=\;j_{0}\text{sin}2\theta_{0},$$

where *j*_0_ is the amplitude of current, so it can be named as ALPGE current. Peng et al. [7] only revealed excitation by single wavelength, and there was not a regular transition corresponding to interband transition. While in this letter, we report the observation of ALPGE and its spectra dependence in an undoped (001)-oriented InGaAs/AlGaAs multiple quantum well (MQW) with *C*_2*v*_ symmetry, which is a good supplement to the in-depth investigation of the spectra dependence of momentum anisotropy of the photoinduced carriers in semiconductors.

## Methods

The sample studied here is an undoped In _0.15_*Ga*_0.85_As/Al _0.3_*Ga*_0.7_As quantum wells (QWs) grown by molecular beam epitaxy. A 200-nm buffer layer is initially deposited on (001) SI-GaAs substrate, followed by ten periods of 100-Å In _0.15_*Ga*_0.85_As/100-Å Al _0.3_*Ga*_0.7_As QWs. Then, a 500-Å Al _0.3_*Ga*_0.7_As layer and 100-Å GaAs cap layer are deposited. The sample is cleaved into a narrow strip along the GaAs $\left[\;1\bar{1}0\right]$ direction with a width of 4 mm and a length of 12 mm, respectively. The geometry has been shown in the inset of Figure 2a, where two ohmic electrodes with a distance of 3 mm were made along *y* direction by indium deposition and annealed at about 420°C in nitrogen atmosphere.

The experimental setup and method are described as follows: A 140-fs, 80-MHz Ti sapphire oscillator with a peak power of the optical pulses of 300 KW serves as the radiation source. As the full width at half maximum (FWHM) of the laser pulse is about 7 nm, the step-length of the spectral scanning should not be too small. In this experiment, the excitation wavelength is tuned from 865 to 1,000 nm with a step-length of 5 nm, which covers both 1hh-1e (the first valence subband of heavy hole to the first conduction) transition and 1lh-1e (the first valence subband of light hole to the first conduction) transition [9]. The incident light goes through a polarizer and a photoelastic modulator (PEM), of which the peak retardation is set to be *λ*/4, to yield a modulated linearly polarized light with a fixed modulating frequency at 100 KHz. The angle between the polarization direction of the incident light and the optical fast axis of the PEM is set to be 45°. The Gaussian profile light beam irradiates vertically on the sample with a diameter of about 2 mm at the perpendicular bisector of the two circle electrodes (see the inset of Figure 2a). After the amplification by a preamplifier, the ALPGE current is collected by a lock-in amplifier through the two circle electrodes. At each light spot, one could get a spectrum dependence of ALPGE current. With the movement of the light spot along the perpendicular bisector of the two circle electrodes, one could get a two-dimentional graph of ALPGE currents as functions of wavelength and light spot, as shown in Figure 2b. As a comparison, the common photocurrent (*I*_*PC*_) is also measured with reference frequency of 220 Hz provided by an optical chopper. The polarization independent light modulated by the optical chopper will induce photoinduced carriers on the plane of the QWs. When the electric field (100 V/cm in this letter) applies on the two circle electrodes, *I*_*PC*_ will be generated, which is collected by the lock-in amplifier, so one can see that *I*_*PC*_ is proportional to the density of the photoinduced carriers. All the experiments are carried out at room temperature.

## Theoretical model

Different from the ordinary LPGE due to asymmetry scattering on phonons, static defects, or other carriers in noncentrosymmetric media, the ALPGE is supposed to due to the optical momentum alignment effect [1,7]. This ALPGE is similar to the so-called surface photogalvanic effect in [10-12] and the edge photogalvanic effect in [13], where the electrical detection of optical momentum alignment is carried out in metals as well as semiconductors. However, optical momentum alignment alone is not enough to induce a net electric current due to the symmetrical distribution of carriers with momenta ***k*** and ***−k*** in optical excitation. It has been believed that the diffuse scattering of the surface or the edge breaks the symmetrical distribution of carriers with opposite ***k***. While in the MQW studied here, the optical absorption corresponds to the inner wells and the size of the sample is much larger than the diameter of the laser beam and can be considered edgeless, thus the surface as well as the edge photogalvanic effect can be neglected in our experiment. It is believed that in our experiments the inhomogeneity of light intensity plays the same role with the surface or the edge for generating a net electric current.

As shown in Figure 1c,d, the distribution of photoinduced carriers has a Gaussian profile due to the Gaussian light beam. During the momentum relaxation time, the carriers will have a very small displacement along their own momentum directions, which is on the scale of the mean free path. Because the carriers with momenta ***k*** and ***−k*** will have opposite displacements, a small spatial separation of the two groups of carriers is generated, which results in a photocurrent proportional to the gradient of carrier density. Since the radius of light spot with about 2 mm is much bigger than the momentum diffusion length, the contribution of the current outside the light spot can be neglected. Therefore, the ALPGE current is decided by the distribution of the light intensity which can be expressed phenomenologically as

(3) $${\text{j}}_{\text{ALPGE}}\;=\;\phi \nabla \text{I(r)},$$

where *ϕ* is a second-rank symmetric tensor relating to the optical momentum alignment effect, *I*(*r*) is the light intensity with a Gaussian distribution, and *r* is the radial direction in the *x*−*y* plane. The carrier distributions in QWs are highly influenced by *k*-linear terms in band structure and transition probabilities [14,15]. For the sake of simplicity, we still use bulk equations of the optical momentum alignment effect in the following simulations. According to [7], *ϕ* can be expressed as

(4) $$\phi =-\frac{2q{\tau^{*}}^{2}\alpha }{\hbar \omega k_{\text{ex}}}\int \delta (k-k_{\text{ex}})g(\theta ,\theta_{0})\mathrm{\upsilon \upsilon dk},$$

where *τ*^∗^ is the effective relaxation time, *α* is the optical absorption coefficient, *ω* is the photon frequency, *υ* is the velocity of carriers, *k*_*ex*_ is the magnitude of the wave vector at the exciting point, and *g*(*θ*,*θ*_0_) is the momentum anisotropy factor. The *g*(*θ*,*θ*_0_) factor describes the probability of an excited carrier’s momentum being along the *θ* direction given the light polarization plane along the *θ*_0_ direction. According to the optical momentum alignment effect theory [4], *g*(*θ*,*θ*_0_) can be expressed as $g(\theta ,\theta_{0})=\frac{3}{2}\;f_{0}\text{si}n^{2}(\theta -\theta_{0})$ for 1hh-1e transition and as $g(\theta ,\theta_{0})=\frac{3}{2}\;f_{0}\;\left[\text{co}s^{2}(\theta -\theta_{0})+1/3\right]$ for 1lh-1e transition, where *f*_0_ is the symmetric part of the the momentum anisotropy factor (see Figure 1a,b). As a result, an anisotropy electric current will be induced around the light spot, which will further generate an observed ALPGE current.

## Results and discussion

In the experiment, the ALPGE currents are measured as a function of the spot location at different wavelengths (near the transitions of 1hh-1e (955 nm) and 1lh-1e (905 nm)). As shown in Figure 2a, the ALPGE currents reverse the sign from the left to the right side, just like a sine curve, which is quiet similar to the anomalous circular photogalvanic effect (ACPGE) current (arising from the reciprocal spin Hall effect) where there is a current swirling over the center of the light spot [16]. However, different from the ACPGE, the ALPGE is derived from an anisotropic distribution of current due to the optical momentum alignment effect. The movement of the anisotropic current along *x* direction will change the amplitude of the ALPGE current. Taking the light spot as a simple geometric point, i.e., *I*(*r*)=*Wδ*(*r*), the detected current between the two electrodes can be expressed as [7]

(5) $$I_{\text{ALPGE}}=\frac{4W\phi_{\text{xy}}}{\mathrm{\pi \sigma R}}\frac{\text{xL}}{{\left(x^{2}+L^{2}\right)}^{2}},$$

where *σ* is the conductivity which is considered to be the same at varied wavelengths with the same light intensity and *R* is the resistance between the two electrodes. As shown in Figure 2a, Equation (5) fits well with the experimental data. The small deviation of experimental data from the fitting line might be attributed to the nonuniformity of the sample. Since the momentum of hole is known to relax very fast, on a longer time scale, one can just take the electron contribution to the current into consideration. Under the effective mass approximation, the second-rank symmetric tensor *ϕ*_*xy*_ is expressed as [7]

(6) $$\phi_{\text{xy}}=\alpha \beta (\theta_{0})\frac{2e{\left(\upsilon_{e}\tau^{*}\right)}^{2}}{\hbar \omega },$$

where $\beta (\theta_{0})=\int g(\theta ,\theta_{0})\mathrm{sin\theta cos\theta d\theta}$ is the momentum anisotropy factor in this experiment (where *θ*_0_ is fixed on 45°) and $\upsilon_{e}=\frac{\hbar k_{\text{ex}}}{m^{*}}$ is the electron velocity. *β*(*θ*_0_) is calculated as

(7) $$\beta (\theta_{0})\;=\;-\frac{3}{8}\pi f_{0}\text{sin}2\theta_{0}$$

for 1hh-1e transition and

(8) $$\beta (\theta_{0})\;=\;\frac{3}{8}\pi f_{0}\text{sin}2\theta_{0}$$

for 1lh-1e transition.

As shown in Figure 2b, the extreme positions are all focused on the location of *x*=±0.87 mm, which indicates that the extreme position of ALPGE current is wavelength independent. When the wavelength is tuned from 895 to 980 nm, the changes of the size of the light spot can be neglected. In fact, the vary of wavelength only changes the density of photoinduced electrons which can be written as $n=\int \frac{\mathrm{\alpha I}(r)}{\hbar \omega }\text{dr}=\frac{\mathrm{\alpha W}}{\hbar \omega }$ and the effective relaxation time *τ*^∗^. Therefore, according to Equations (5) and (6), the vary of wavelength only affects the amplitude of the ALPGE current but does not change the extreme position.

**Figure 3:**
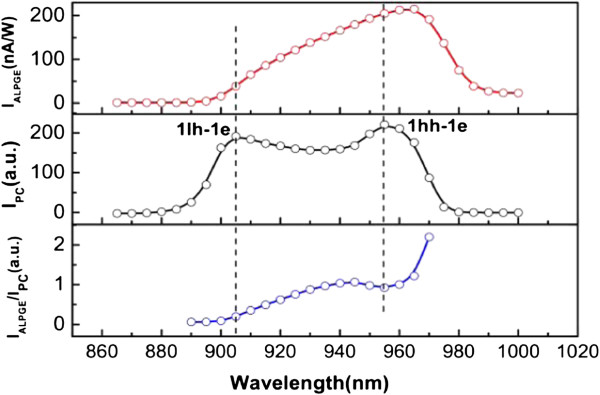
**The spectrum of ALPGE and PC currents.** The light spot is fixed on the position of *x*=+1 mm. **(a)** The red empty circles denote the ALPGE current as a function of excitation wavelength. **(b)** The black empty circles denote the common photoinduced current as a function of excitation wavelength. The external electric field applied on the two circle electrodes is 100 V/cm. **(c)** The blue empty circles denote the ALPGE current normalized by the common photoinduced current. All of the solid lines are for ease of viewing.

Figure 3a shows the ALPGE current as a function of wavelength when the light spot is fixed on the position of *x*=+1 mm. From Equations (7) and (8), we can see that in both situations *β*(*θ*_0_) are equal in size but different in signs. Therefore, one should observe that the ALPGE current reverses the sign when the optical interband transition changes between 1lh-1e and 1hh-1e. However, we do not observe obvious opposite in the sign, which is probably due to the strong band mixing [15], that is, the signal corresponding to 1lh-1e is contributed from not only the 1lh-1e transition but also the 1hh-1e transition. In other words, signal corresponding to 1lh-1e is small, and the current corresponding to the so-called 1lh-1e marked in Figures 2 and 3 is actually the combined action of 1lh-1e and 1hh-1e. As a comparison, the common photoinduced current is also measured under the same conditions, as shown in Figure 3b. According to Ohm’s law, the common photoinduced current can be written as

(9) $${\text{I}}_{\text{PC}}\;=\;n\text{e}\mu_{n}\text{E},$$

where $\mu_{n}=\frac{e\tau_{p}}{m^{*}}$ is the mobility of electrons and *E* is the external electric field. One can surprisingly find that the two curve shapes in Figure 3a,b are quite different. To eliminate the difference of the nonequilibrium electron’s density at different wavelengths, we normalize the ALPGE current with the common photoinduced current, because the common photoinduced current is proportional to the density of photoinduced nonequilibrium electrons. And the normalized ALPGE current is shown in Figure 3c. According to Equations (5) and (9), the normalized *I*_*ALPGE*_ (see Figure 3c) can be simplified as

(10) $$\frac{I_{\text{ALPGE}}}{I_{\text{PC}}}\propto \frac{{\tau^{*}}^{2}}{\tau_{p}}.$$

The contribution to *τ*^∗^ contains three parts: the momentum relaxation time *τ*_*p*_, the energy relaxation time *τ*_*ε*_ which describes the inelastic processes due to the scattering of longitudinal optical phonon, and the carrier lifetime *τ*_0_ which describes the carrier recombination, i.e., ${\tau^{*}}^{-1}=\tau_{0}^{-1}+\tau_{\varepsilon }^{-1}+\tau_{p}^{-1}$. Given the average mobility of the photoinduced electrons to be 8,000 *cm*^2^/(*V*·*s*) and the effective mass of electrons to be 0.06 *m*_0_ (where *m*_0_ is the mass of electron in a vacuum), the momentum relaxation time *τ*_*p*_ is estimated to be 0.27 ps, which would be smaller at higher excited states. Since *τ*_0_∼1*ns*[17], *τ*_*ε*_∼0.15−7*ps*[18], one can see that *τ*^∗^ is mainly determined by the momentum relaxation time *τ*_*p*_ and the energy relaxation time *τ*_*ε*_. The decrease of the normalized *I*_*ALPGE*_ can be attributed to the faster momentum and energy relaxation at smaller excitation wavelengths. We have also testified that the ALPGE current still exist in undoped GaAs/AlGaAs QWs; however, we observed no obvious ALPGE current in undoped bulk GaAs. What is more, the ALPGE current in this strained InGaAs/AlGaAs QWs is much stronger than that in undoped GaAs/AlGaAs QWs. Thus, we believe that the ALPGE is a common phenomenon in low-dimensional semiconductors, and the ALPGE is probably stronger in strained semiconductors.

## Conclusions

The spectra dependence of ALPGE at normal incidence in undoped InGaAs/AlGaAs multiple quantum well has been studied. This effect is believed to originate from the optical momentum alignment effect and requires an inhomogeneous light illumination. We found that the spot location with the maximum ALPGE current is wavelength independent. We do not observe obvious opposite in the sign for 1hh-1e and 1lh-1e transitions, which is probably due to the fact that the detected current is mainly derived from 1hh-1e transition. And the normalized ALPGE current decreases at smaller wavelengths, which can be attributed to the sharp decrease of the momentum and energy relaxation time. This high sensitivity of electrical measurements proves to be an effective method for detecting the momentum anisotropy of photoinduced carriers and band coupling.

## Competing interests

The authors declare that they have no competing interests.

## Authors’ contributions

LZ conducted the experiments and wrote the paper. LZ and YC designed the experiments and performed the sample fabrications. All authors contributed through scientific discussions and read and approved the final manuscript.
